# Humans have already increased the risk of major disruptions to Pacific rainfall

**DOI:** 10.1038/ncomms14368

**Published:** 2017-02-08

**Authors:** Scott B. Power, François P. D. Delage, Christine T. Y. Chung, Hua Ye, Bradley F. Murphy

**Affiliations:** 1 Bureau of Meteorology, Docklands 3008, Victoria, Australia

## Abstract

Intermittent disruptions to rainfall patterns and intensity over the Pacific Ocean lasting up to ∼ 1 year have major impacts on severe weather, agricultural production, ecosystems, and disease within the Pacific, and in many countries beyond. The frequency with which major disruptions to Pacific rainfall occur has been projected to increase over the 21st century, in response to global warming caused by large 21st century greenhouse gas emissions. Here we use the latest generation of climate models to show that humans may have contributed to the major disruption that occurred in the real world during the late 20th century. We demonstrate that although marked and sustained reductions in 21st century anthropogenic greenhouse gas emissions can greatly moderate the likelihood of major disruption, elevated risk of occurrence appears locked in now, and for at least the remainder of the 21st century.

## Abstract (web summary)

Intermittent rainfall changes over the Pacific Ocean can profoundly disrupt lives and ecosystems in many locations. Here, the authors show that the risk of such changes has increased, and that the risk could – even with a reduction in greenhouse gas emissions - remain elevated for decades to come.

Year-to-year disruptions to seasonal rainfall patterns and rainfall amounts over the Pacific Ocean are primarily driven by the El Niño-Southern Oscillation (ENSO), which is a naturally occurring phenomenon centred in the tropical Pacific^1^,^2^. These disruptions have major impacts on severe weather, agricultural production, ecosystems, and disease in the Pacific, and in many countries beyond^1^,^3^,^4^,^5^,^6^,^7^,^8^,^9^,^10^,^11^,^12^,^13^. Recent research^14^,^15^,^16^ concluded that the frequency of disruptions to Pacific rainfall over the 21st century associated with ENSO will be much larger than it was during the 20th century. These studies focused on changes over the entire 21st century relative to the entire 20th century, under high greenhouse gas scenarios (SRES A2) (ref. 17), RCP8.5 (refs 18, 19)).

Two important questions which have not been addressed previously are: “Has the risk (that is, likelihood) of major disruption driven by year-to-year rainfall variability already increased?”, and “Can the projected 21st century increase in risk be avoided or moderated by substantial and sustained reductions in global greenhouse gas emissions?” The first question is partially motivated by recent research indicating that the atmosphere overlying the Pacific Ocean has already warmed to levels that are unprecedented in the historical record^20^. The second question arises because governments from around the world have recently agreed to markedly reduce global greenhouse gas emissions over coming decades. But will these cuts be sufficient to prevent a human-forced increase in the risk of major disruption?

To address these questions, we examine disruption in an ensemble of CMIP5 models spanning the pre-industrial era to the late 21st century. We find that the risk of major rainfall disruptions has already increased, and that the risk will remain elevated for the remainder of the 21st century, even if marked and sustained reductions in global greenhouse gas emissions are made. The increase in disruption risk is caused by anthropogenic warming that drives an increase in the frequency of ENSO events and an intensification of ENSO-driven rainfall anomalies in the central-eastern equatorial Pacific.

## Results

### Measuring disruptions

We define direct measures of disruption to precipitation over the entire region of interest (that is, 140° E–240° E, 25°S–15° N). This contrasts with a previous study that used a proxy measure of disruption based on rainfall amount at a single location^14^. We will use two different measures of disruption: the spatial correlation coefficient (*R*); and the root-mean-squared difference (*D*), between seasonal (December–January–Februrary) and long-term (multi-decadal) average rainfall patterns. *R* in any given year is equal to the (spatial) correlation coefficient between average seasonal rainfall in that year with average seasonal rainfall over all years in the multi-decadal period in which that year falls. *R* is therefore a measure of the degree to which the spatial pattern of rainfall in individual years deviates from the climatological pattern. In a similar fashion, *D* is a measure of the magnitude of rainfall anomalies in individual years, relative to the climatological pattern over the same region. In the following we will refer to Ω_R_, Ω_D_ and Ω, where Ω_R_ is the frequency of major disruption defined in terms of *R*, Ω_D_ is the frequency of major disruption defined in terms of *D*, and Ω is used to refer to Ω_R_ and Ω_D_ collectively.

Fourteen multi-decadal periods are considered: 10 during the pre-industrial period, and one each during the early 20th century (E20C), the late 20th century (L20C), the early 21st century (E21C) and the late 21st century (L21C). Furter details on why these periods are chosen is given in ‘Methods' section.

### Major disruptions

**Figure 1:**
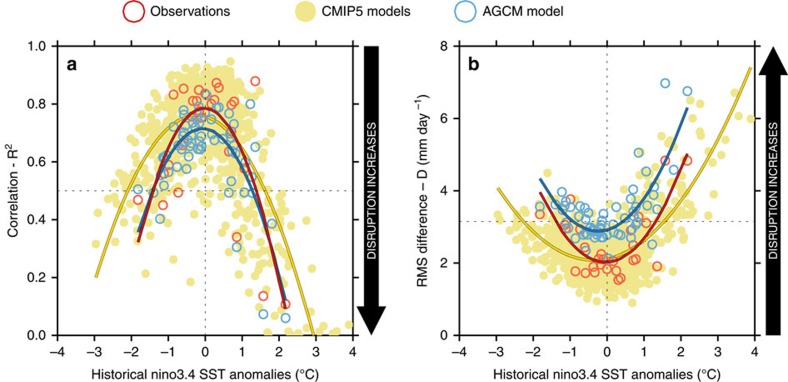
Scatter plots showing measures of disruption versus the NINO3.4 SST anomaly. (**a**) *R*^*2*^ and (**b**) *D*. *R* is the correlation coefficient between the rainfall map in a given year with the map of rainfall averaged over a longer reference period in which the individual year falls. *D* is the root-mean-squared difference between seasonal and long-term (multi-decadal) average rainfall patterns. *R* and *D* are calculated over the domain 140° E–240° E, 25° S–15° N. Observations (unfilled red circles^21^,^22^), with reference period 1979–2013. The coupled climate models (small filled mustard-coloured dots), with reference period L20C (that is, 1957–1992). AGCM (unfilled blue circles) with reference period 1951–2013. AGCM values given are the averages of values in a 10-member ensemble. Quadratics-of-best-fit are also shown using the same colour scheme. Note that El Niño events have positive NINO3.4 SST anomalies, and so they appear to the right of the *y* axis in both panels, while La Niña events appear to the left. The greater the disruption the smaller *R* becomes, and the larger *D* becomes. All values are for December–February.

The relationship between *R*^*2*^ and NINO3.4 sea-surface temperature (SST) anomalies is presented in Fig. 1a for the observations^21^,^22^, coupled climate models^23^ under 20th century forcing, and an atmospheric general circulation model (AGCM)^24^,^25^,^26^ forced using SSTs observed from 1951 to 2013 (see ‘Methods' section). The results indicate that the frequency and strength of disruptions tends to increase as the magnitude of NINO3.4 anomalies increase, and that major disruptions (defined here to occur when *R*^2^<0.5) can occur during both El Niño and La Niña years—the two extreme phases of ENSO. Under this definition the observations exhibited major disturbances during the El Niño years of 1991/92, 1982/1983 and 1997/98, and during the La Niña years of 1988/1989, 2000/2001 and 2010/2011. Similar results are obtained if we define major disruption in terms of *D* (Fig. 1b, *D*>3.1 mm day^−1^, which is chosen so that Ω_D_ and Ω_R_ have a similar value in the pre-industrial period (1.1 events per decade)). These thresholds are based on an analysis of the 24 CMIP5 models selected which had at least 500 years available under pre-industrial forcing (identified in Supplementary Table 1). The strong asymmetry evident in observational, coupled model and AGCM data indicates that much greater disruption can occur during El Niño years than during La Niña years.

When a major disturbance occurs during El Niño years (Supplementary Fig. 1a), rainfall tends to extend further east along the equator, there tends to be a reduction in rainfall in the western Pacific, and both the Intertropical Convergence Zone and the South Pacific Convergence Zone tend to move equatorward^6^,^7^,^24^,^26^,^27^,^28^,^29^. While major disturbances during La Niña years tend to exhibit the opposite of these features^6^,^7^,^25^,^27^,^28^,^29^ asymmetries in rainfall anomalies are clearly evident^23^ (Supplementary Fig. 1b). The models (Supplementary Fig. 1c and d) appear to do a reasonable job in capturing the observed behaviour (Supplementary Fig. 1a and b).

### Changes in the frequency of major disruptions

**Figure 2:**
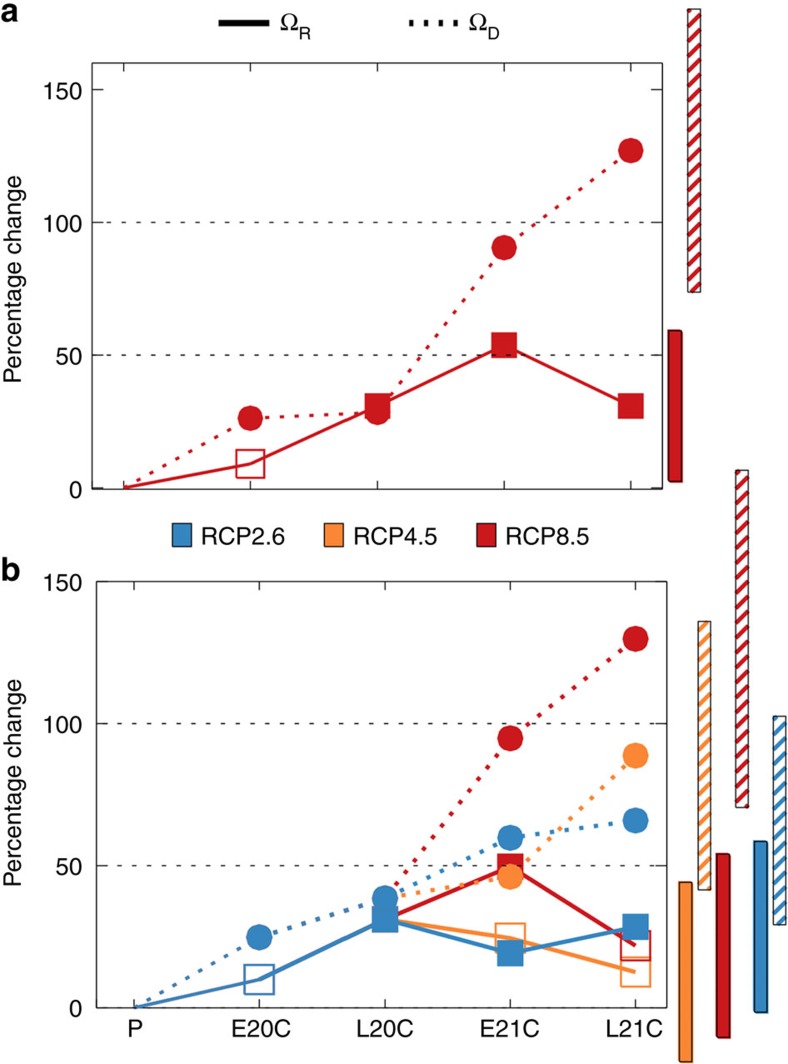
Percentage change in the frequency of major disruptions in the twentieth and twenty-first centuries. E20C, L20C, E21C and L21C frequency changes relative to the pre-industrial period. (**a**) Twenty-first century values under RCP8.5 only. 24 models. (**b**) As in **a** but twenty-first century percentage changes are provided for three different scenarios: RCP2.6 (blue), RCP4.5 (orange) and RCP8.5 (red). The results in **b** are based on changes obtained from the 20 models that were forced with all three scenarios (see ‘Methods'). Dashed lines and circles indicate percentage changes in Ω_D_, solid lines and squares indicate percentage changes in Ω_R_, both relative to their pre-industrial values. Filled circles and squares indicates that *P*_rank_*<*0.1. Bars indicate the 90% confidence interval of the multi-model mean (MMM) change for L21C.

Relative to the pre-industrial period, there is a 10% increase in the multi-model mean (MMM) of Ω_R,_ MMM(Ω_R_), in E20C (Fig. 2a, Supplementary Table 2), that is, a 10% increase in the frequency of major disruption to the spatial rainfall pattern (when *R*^*2*^ falls below 0.5). In the E20C period, 14 of the 24 models show an increase in Ω_R_, 9 models show decreases, and one model shows no change (*P* value=0.21; Supplementary Table 2). There is also a 31% increase in MMM(Ω_R_) in L20C (Fig. 2a), with 17 increases and 5 decreases (*P* value=0.01), a 54% increase in E21C with 19 increases and 4 decreases (*P* value*<*0.01), and a 31% increase in L21C with 14 increases and 8 decreases (*P* value=0.15). These 21st century increases occur under the 21st century scenario with the highest greenhouse gas increases (RCP8.5, Fig. 2a), with the clearest indication of change occuring for E21C. It is interesting that the change in E21C is larger than for L21C. We will return to this issue later.

The corresponding changes in Ω_D_ are 26, 28, 90 and 126%, for E20C, L20C, E21C and L21C respectively (Fig. 2a). The associated *P* values are 0.03, 0.03, <0.01, and 0.01 respectively (Supplementary Table 3). The 21st century figures for Ω_D_ are larger than the corresponding figures for Ω_R_ (that is, 54 and 31%). This indicates that 21st century global warming has a greater impact on disruptions to the magnitude of year-to-year variability than it does on the spatial structure of the variability.

Additional analysis indicates that the increases in Ω arise from increases in the frequency of major disruptions during both extreme phases of ENSO. For example, there is a 21% increase in Ω_R_ during El Niño years (*P* value=0.03), and a 91% increase in La Niña years (*P* value=0.05), in E21C relative to the pre-industrial period.

### Factors responsible for the increase in major disruptions

We will show below that the increase in MMM(Ω) has contributions from: (i) an increase in the frequency of El Niño and La Niña events; and (ii) an increase in precipitation anomalies arising from a nonlinear interaction between unchanged ENSO-driven SST anomalies and background (global) warming^5^,^24^,^25^,^26^,^27^,^28^. The positive contributions from nonlinearity can be reinforced or partially offset in models, depending on what happens to the magnitude of ENSO-driven SST variability in each model.

### Changes in ENSO frequency

There is a tendency for the frequency of both El Niño and La Niña events—defined in terms of SST variability (see ‘Methods' section)—to increase. For example, the MMM frequency of La Niña events during the pre-industrial period is 2.3 per decade, and this figure increases by 4% during E20C, 10% during L20C, and by 22% during E21C and 9% during L21C under the RCP8.5 scenario. The MMM frequency of El Niño events during the pre-industrial period is 2.2 per decade, and this figure increases by 2% in E20C, 12% in L20C, and by 22 and 7% in E21C and L21C, respectively (both under RCP8.5). Note that only the increases in L20C and E21C are statistically significant.

### Precipitation anomaly increases

A useful and important indicator of rainfall variability and disruption in the Pacific is the amount of rainfall received in the central-eastern equatorial Pacific^5^,^14^. By the end of the 20th century, the magnitude of precipitation anomalies (relative to a new, background state) in the central-eastern Pacific during El Niño and La Niña events tends to increase with time, as the world warms up. This intensification of ENSO-driven rainfall variability will increase the likelihood that a given El Niño or La Niña event will cause major disruption. For example, during La Niña events, 33% of models show an increase in the magnitude of rainfall anomalies averaged over the NINO3.4 region during E20C, 62% during L20C, 71% during E21C and 83% during L21C. The corresponding increase during El Niño events are 54%, 67%, 88% and 71%, respectively.

Modelled changes in the magnitude of NINO3.4 SST anomalies during ENSO events are varied and do not exhibit the degree of consistency that the modelled precipitation changes exhibit. For example, during El Niño events, only 58% of models show an increase in the magnitude of the NINO3.4 SST anomaly in E20C under RCP8.5. Correspondingly, in L20C, E21C, and L21C, only 63, 50 and 38% of models show an increase. During La Niña events 71% of models actually show a decrease in the magnitude of the NINO3.4 SST anomalies in E20C, while increases of 50, 58, and 67% are evident for the later periods (with E21C and L21C values again obtained under the RCP8.5 scenario).

**Figure 3:**
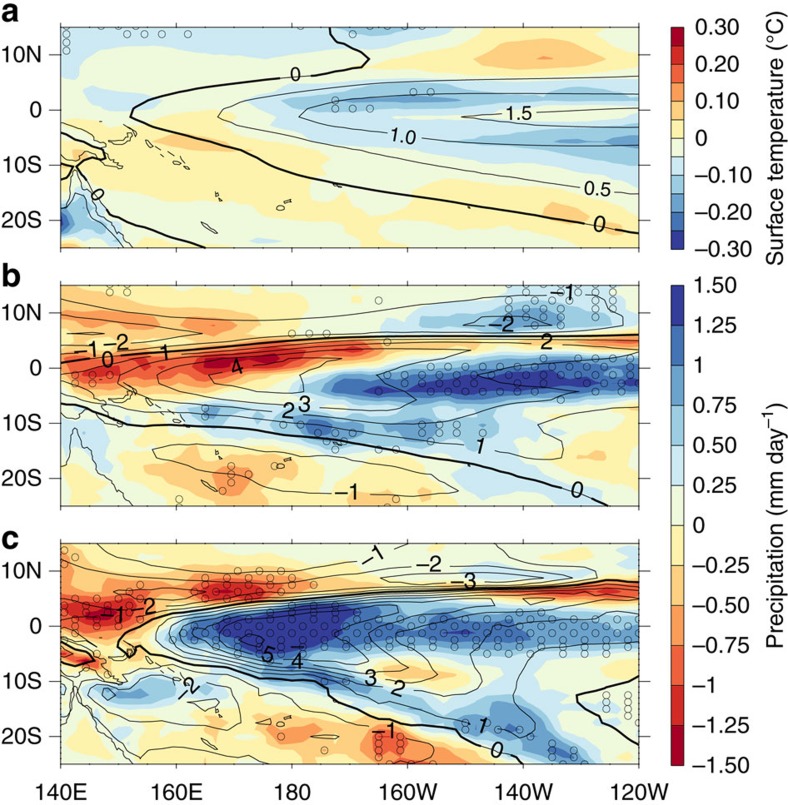
Changes in El Niño-driven surface temperature and rainfall anomalies. Changes in (**a**) surface temperature anomalies, and in precipitation anomalies in both (**b**) the climate models and (**c**) the AGCM. There is no change at all in the ENSO-driven SST anomalies used to generate **c**. Climate models: L21C relative to pre-industrial. AGCM: L21C relative to L20C. Maps were generated using Interactive Data Language (The Interactive Data Language (Version 8.2.3) [Software]. (2013). IDL, Exelis Visual Information Solutions, Inc., is a subsidiary of the Harris Corporation. http://www.harrisgeospatial.com/).

Multiple pieces of evidence support the importance of an increase in precipitation anomalies arising from a nonlinear interaction between unchanged ENSO-driven SST anomalies and background warming. Here, nonlinearity refers to the fact that exactly the same ENSO SST anomaly, when combined with background warming, can result in a different precipitation anomaly (measured relative to a new climatological precipitation value). The first piece of evidence is given in Fig. 3, which shows the change in both the SST anomaly (Fig. 3a) and precipitation anomaly (Fig. 3b) during El Niño events, between the pre-industrial period and L21C. There is little agreement among all models on the sign of change in SST anomalies (that is, lack of stippling in Fig. 3a). Despite this there is agreement on an enhancement of the rainfall signal.

Additional evidence for intensification of rainfall anomalies through nonlinearity is provided by the similarity between the patterns of change in precipitation anomalies obtained from the SST-forced AGCM experiments (Fig. 3c)—in which there is no change at all in ENSO-driven SST anomalies—and the MMM pattern of change in the coupled climate models (Fig. 3b).

It is reassuring to note the magnitude of the nonlinear reinforcement of El Niño-driven rainfall anomalies over the NINO3.4 region in the coupled models and AGCM have a similar scale in Fig. 3 (∼0.61 mm day^−1^ (models) and 0.7 mm day^−1^ (AGCM)). A breakdown of the moisture budget in the AGCM, described in ‘Methods', shows that the changes in rainfall primarily arise from changes in the mean circulation dynamics (Supplementary Fig. 2). These changes are partially offset by contributions from the covariant component comprising transient eddy and surface terms, and are weakly enhanced by a contribution from a thermodynamic component which reflects an increase in the available moisture.

An enhancement of the La Niña-driven rainfall response in the climate models is also evident (Supplementary Fig. 3b). This occurs despite there being very little consistency in the change in La Niña-driven SST variability (Supplementary Fig. 3a). This is also consistent with the impact of global warming on La Niña-driven rainfall responses in the AGCM in which there are no changes in the La Niña-driven SST anomalies at all (Supplementary Fig. 3c).

Our results are consistent with earlier research^5^,^24^,^25^,^26^,^30^,^31^, which collectively indicates that changes in ENSO-driven precipitation variability can be explained in terms of changes in four factors: (i) mean-state moisture content, (ii) the amplitude of ENSO-driven SST variability, (iii) spatially dependent changes in mean-state SST and (iv) in the structure of ENSO-driven SST variability. One of these studies^31^ highlighted the importance of the contrast between mean-state SST changes in the tropical Pacific and changes in mean-state SST throughout the tropics.

**Figure 4:**
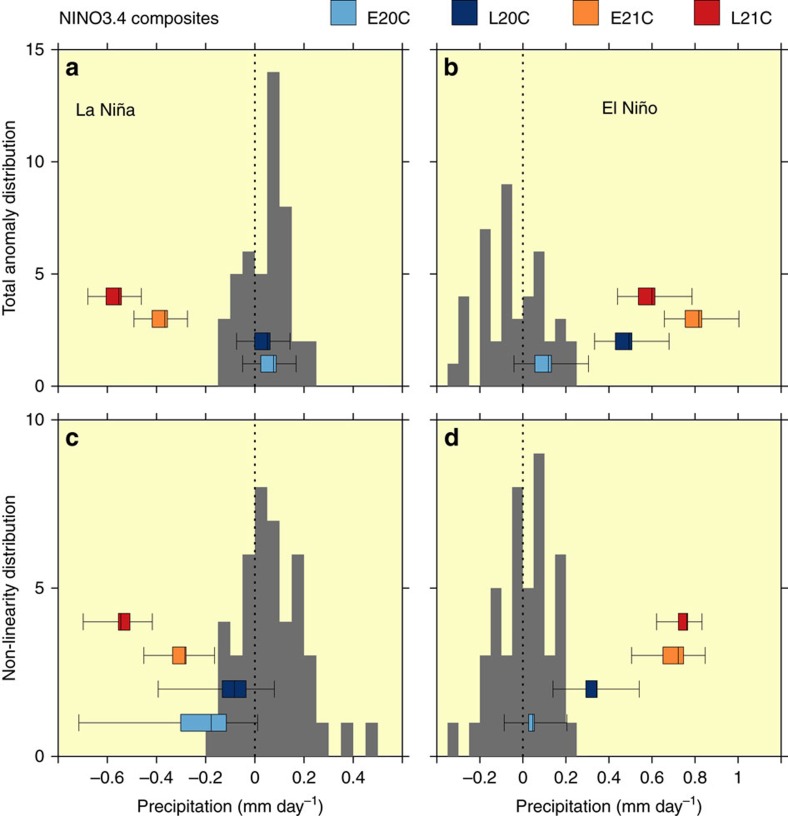
Relative frequency distributions of changes in NINO3.4 region. (**a**,**b**) rainfall anomalies and (**c**,**d**) nonlinear contributions. All changes are relative to 10 different pre-industrial periods. The grey bars represent the distribution of changes between the 10 pre-industrial periods. The grey bars therefore represent changes that can arise from internal climate variability alone. The boxplots represent the changes for E20C (light blue), L21C (dark blue), E21C (orange), L21C (red), relative to the 10 pre-industrial values. The whiskers indicate the minimum and the maximum change, the boxplot the 25th and 75th percentiles, and the median is indicated by the vertical line in the boxes. The approach used to estimate the nonlinear contribution is described in ‘Methods'.

Estimates of the contribution of nonlinearity to changes in ENSO rainfall anomalies are given in Fig. 4. Figure 4a (La Niña) and Fig. 4b (El Niño) give the modelled relative frequency distributions of changes in NINO3.4 rainfall anomalies, while Fig. 4c,d give the modelled, relative frequency distributions of the nonlinear contribution to the change in the corresponding rainfall anomalies above. The method used to estimate nonlinearity is described in ‘Methods'. Changes arising from internal variability alone are estimated by differences between each multi-decadal pre-industrial period with every other multi-decadal pre-industrial period (grey bars). For El Niño (Fig. 4d), nonlinearity makes a positive (enhancing) contribution in all of the 20th and 21st century periods (that is, E20C, L20C, E21C, L21C). This contribution is largest in the 21st century (that is, in E21C and L21C), and smallest in E20C. In fact the change in E20C is very small, and is typically within the range of internal variability. This is not the case for the other three periods. Nonlinearity in L20C, E21C and L21C is larger than E20C, and is beyond the internal variability range depicted. This indicates that the changes in El Niño rainfall anomalies are at least partially caused by external forcing during L20C, E21C and L21C.

For La Niña, the nonlinear contribution tends to be negative for all 20th and 21st century periods. However, only the 21st century changes in nonlinearity (Fig. 4c) are very largely outside the internal variability range depicted. As rainfall tends to decline in the NINO3.4 box during pre-industrial La Niña events, the negative value of the 21st century nonlinear contributions again indicates that nonlinearity acts to enhance the ENSO-driven rainfall anomaly. This nonlinear enhancement is consistent with, but extends, earlier research on El Niño-driven rainfall changes in coupled models^5^ and ENSO-driven changes in an AGCM^24^,^25^,^26^.

One puzzle, which we have not yet addressed, is why there is a decrease in Ω_R_ from E21C to L21C under RCP8.5 (Fig. 2a, for example MMM(ΔΩ_R_)=−0.3 events/decade). This is, at least in part, due to a decline in the magnitude of El Niño-driven SST anomalies in most models between these two periods (Supplementary Fig. 5), consistent with earlier research^32^. Such declines oppose, and evidently overcome, the nonlinear enhancement of precipitation anomalies in L21C, relative to E21C (Fig. 4).

### Impact of reducing 21st century global emissions

We have so far restricted the analysis of 21st century changes to those that occur under the highest 21st emissions scenario—RCP8.5. The changes in Ω_R_ and Ω_D_, under all three scenarios considered—RCP2.6, RCP4.5 and RCP8.5—are presented in Fig. 2b and Supplementary Tables 2 and 3 for the 20 models which have results for all three scenarios. The frequency of major disruption increases relative to pre-industrial levels under all three scenarios. The increases tend to be largest under RCP8.5 and smallest under RCP2.6. The 21st century increases in Ω_D_ under RCP2.6 are consistent with a previous study^33^ indicating an increase in the magnitude of variability in precipitation anomalies in the central-eastern Pacific under this scenario.

## Discussion

Four important conclusions can be drawn from the results. First, the risk of major disruption to rainfall patterns and rainfall intensity had already increased by the end of the 20th century (see for example L20C in Fig. 2a or Supplementary Tables 2 and 3). This means, for example, that some of the disruption actually witnessed in the real world during L20C might have been partially due to anthropogenic increases in greenhouse gas concentrations that had already occurred by that time^34^,^35^. Second, the risk is elevated today (for example, Fig. 2, E21C). Third, further increases in the risk of major disruption during the remainder of the 21st century can be strongly moderated if major and sustained cuts to global emissions of greenhouse gases are made—as they are in RCP2.6. However, the fourth and final point, is that elevated risk appears locked in for at least the remainder of the 21st century. This is true even if global action is successful in restricting future anthropogenic climate change to RCP2.6 levels—levels which may keep global warming below 2 °C relative to the latter half of the 19th century^34^,^35^.

## Methods

### Models and scenarios used

Twenty-four models forced using both historical forcing (HIST) and forcing under the RCP2.6, RCP4.5 and RCP8.5 scenarios from the CMIP5 archive^23^ are used in this investigation. Models were selected when at least 500 years of pre-industrial runs were available. All coupled climate models and the observations were re-gridded to a 1.5° latitude/1.5° longitude grid before analysis. See Supplementary Table 1 for a list of the models used and the forcing applied. One subset of 20 models is also considered in relation to Fig. 2b: the 20 models for which simulations under RCP2.6 forcing are also available. RCP8.5 represents a scenario in which there are very high greenhouse gas emissions during the 21st century, RCP2.6 represents a stringent mitigation scenario in which strong and sustained cuts are made to global greenhouse gas emissions during the 21st century , while RCP4.5 is an intermediate scenario. RCP2.6 results in global warming that is likely to be in the range of ∼0.9–2.3 K (relative to the latter half of the 19th century) in the late 21st century^34^,^35^.

### Measuring disruption

*R* during the E20C, for example, is given by *R*(*t*)*=*correl(precip(Φ*,λ,t*), *μ*_E20C_(Φ, *λ*)), where correl is the (spatial) correlation coefficient between the two variables in brackets, precip(Φ*,λ,t*) is the precipitation in an individual season during E20C, *μ*_E20C_(Φ*,λ*) is the seasonal average of precipitation for E20C, Φ is the latitude, *λ* is the longitude and *t* is time. Similarly *R* during L21C is given by *R*(*t*)*=*correl(precip(Φ*,λ,t*), *μ*_L21C_(Φ,λ)), where precip(Φ*,λ,t*) is the precipitation in an individual season during L21C and *μ*_L21C_(Φ, *λ*) is the seasonal average of precipitation for L21C. Similar formulae apply for *D*. The domain used to calculate *R* and *D* is 140° E–240° E, 25° S–15° N. For the observations we use rainfall averaged over the period 1979–2013. For the coupled climate models we use 10 36-year periods under pre-industrial conditions and four later periods: E20C (early 20th century); L20C (late 20th century); early 21st century (E21C); and L20C (late 21st century). The last two were examined under three different scenarios (RCP2.6, RCP4.5 and RCP8.5). In the AGCM experiments we use the period 1951–2013. The impact of global warming on the AGCM is obtained using observed SSTs from the same period, but with global warming added to the SSTs, in conjunction with increases in atmospheric greenhouse gas concentrations. See below for additional details on the AGCM.

### Defining El Niño and La Niña years

To define ENSO years in the presence of a mean-state that is changing in response to external forcing, a spectral filter was used to eliminate climate variability and changes with periods longer than 13 years^5^. EOF analysis^36^ was used to extract the first ENSO pattern in the resulting interannual surface temperature of every model. The EOF analysis of surface air temperature was performed on June–December averages. The periods used for filtering are 1–50, 51–100, 101–150, 151–200, 201–250, 251–300, 301–350, 351–400, 401–450, 451–500, 1900–1949, 1950–1999, 2006–2040 and 2050–2099. Here 1–500 refer to years under pre-industrial forcing, other figures to actual years. The results presented in this paper are based on these periods, after removing the first and last seven years from each period to avoid possible near-end issues associated with spectral filtering.

The magnitude and sign of the time-series associated with EOF1(ST) in each model, *E*(*t*) say, was used to classify years as El Niño, La Niña, or neutral years using *E*>0.8*σ*, *E*<−0.8*σ* or −0.8*σ*≤*E*≤+0.8*σ*, respectively. Ten 36-year periods under pre-industrial conditions were used to estimate *σ* for each model. Here *σ* is the mean s.d. of *E*(*t*) in each model and *t* is time.

### Measuring change and internal variability in Ω

Models with at least 500 years of pre-industrial simulation were selected and the analysis was performed on 10 36-year periods (*P*_*i*_), each within a different 50-year period. We then compared the results to the four 36-year periods of the 20th and 21st centuries (E20C, L20C, E21C, L21C). To illustrate the method here, we present the computation for the metric Ω_R_, for the pre-industrial (*P*_i_) and early 20th century (E20C) periods only.


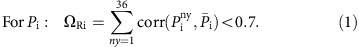



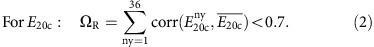


Here *P* is precipitation, ny is the yearly index and 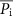 is the average precipitation for the 36-year period of interest.

The internal variability of Ω_R_ for each model is estimated by the variability evident between the 10 different pre-industrial segments. The average value of Ω_R_ over the pre-industrial period is obtained from averaging over all 10 pre-industrial sub-periods. The change in Ω_R_ between the pre-industrial period and E20C is given relative to the average value over the 10 different pre-industrial sub-periods. Similar formulae apply for Ω_D_, and for the periods L20C, E21C and L21C.

### The AGCM

The ACCESS 1.0 AGCM^24^,^25^,^26^ was forced with observed SSTs over the period 1951–2013. Ten ensemble members were generated, each with different initial conditions but the same SSTs. Integrations (10) were then repeated, but with background warming of SSTs in conjunction with higher greenhouse gas concentrations. The warming pattern represents the MMM late 21st century warming of climate models under the SRES A2 scenario^24^.

### Decomposition of precipitation anomaly changes in the AGCM

The precipitation anomaly changes that occur in response to the imposed warming and atmospheric composition changes are decomposed into thermodynamic (TH), dynamic (MCD), covariant (COV) and evaporative (E) components. These terms are calculated using a simplified version^24^ of a method described previously^37^.

### Statistical significance

In the figures we use *P* values based on probabilities from a Binomial Distribution. To estimate the *P* values for the change in Ω_R_ in E20C, for example, a set of eleven Ω_R_ values is formed for each model, using the Ω_R_ value for E20C and all 10 pre-industrial values. For each model, the rank of the E20C value in this eleven-member set, Rank_E20C_ say, is then determined. If Rank_E20C_ is greater than eight it is considered a success, if not, it is considered a failure. This is repeated for each model. The resulting *P* value is then estimated using a binomial distribution with *N*=24 models, and *p*=probability of success=3/11. The resulting *P* values are given in columns labelled *P*_rank_ in Supplementary Tables 2 and 3.

As a test on the robustness of the ranking method used in Supplementary Tables 2 and 3, we use an additional method. This method is also based on a binomial distribution^38^. If *i* models show an increase in Ω, *j* models show no change and *k* show a decrease, then the *P* value is estimated by the probability, Pr, of having *M* successes with *N* trials, where *N=i+j+k.* If *j* is even then *M=i+j*/2. If *j* is odd, then the *P* value is estimated by (Pr_1_*+*Pr_2_)/2. Here Pr_1_ is the probability of *M* successes with *N* trials, where *N* is unchanged and *M=*(*j−1*)/2, and Pr_2_ is the probability of *M* successes with *N* trials, where *N* is unchanged and *M*=(*j+*1)/2. The resulting *P* values are given in columns labelled *P*_sign_ in Supplementary Tables 2 and 3.

### Estimating nonlinearity in precipitation anomaly changes

The nonlinear contribution to the changes in El Niño NINO3.4 precipitation anomalies is based on the relationship between changes in NINO3.4 precipitation and SST changes relative to the pre-industrial period in each model. The line-of-best-fit for the changes in all of the models (with precipitation change on the *y* axis and SST change on the *x* axis—see Supplementary Fig. 4) was then determined for each period (that is, E20C and so on), and each scenario. The *y*-intercept indicates the change in precipitation anomaly that occurs in the absence of any change in SST anomaly. This was repeated using all 10 pre-industrial sub-periods.

### Data availability

The CMIP5 data are available at http://cmip-pcmdi.llnl.gov/cmip5/availability.html.

### Code availability

The code associated with this paper is available on request from S.P.

## Additional information

**How to cite this article:** Power, S. B. *et al*. Humans have already increased the risk of major disruptions to Pacific rainfall. *Nat. Commun.* **8,** 14368 doi: 10.1038/ncomms14368 (2017).

**Publisher's note:** Springer Nature remains neutral with regard to jurisdictional claims in published maps and institutional affiliations.

## Supplementary Material

Supplementary Information Supplementary Figures and Supplementary Tables
