# Supplementary material for: Humans have already increased the risk of major disruptions to Pacific rainfall
Source: Nat Commun. 2017 Feb 8;8:14368. doi: 10.1038/ncomms14368 (PMC5309739; doi:10.1038/ncomms14368)
Supplement: Supplementary Information — Supplementary Figures and Supplementary Tables [file ncomms14368-s1.pdf]

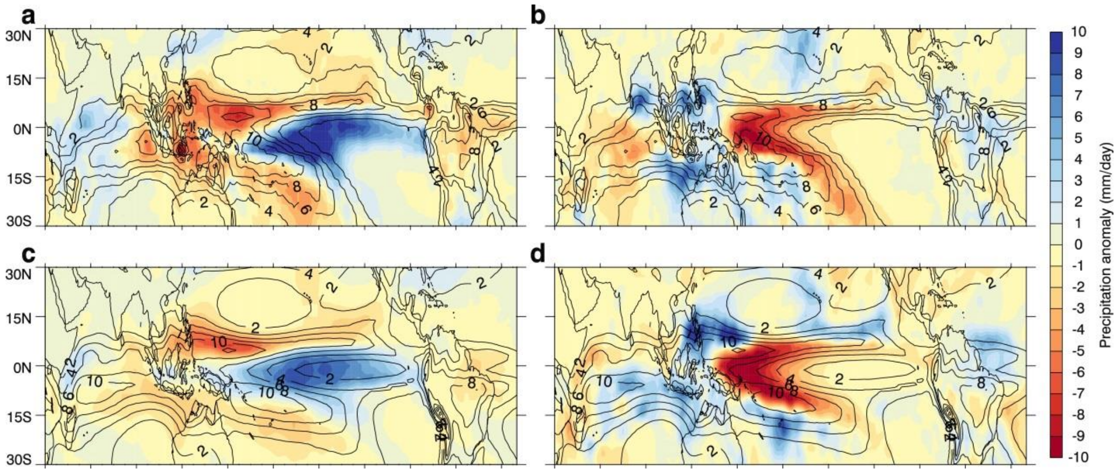

Supplementary Figure 1 | Maps showing composite of rainfall anomalies during major disturbances during El Niño and La Niña years. Observations (*21, 22*): **a,** El Niño, **b,** La Niña. Twenty-four coupled models: **c,** El Niño, **d,** La Niña. Contours: 20th century mean rainfall. Shading: Composites during major disruptions. Maps were generated using IDL Version 8.2.3 (see caption to Figure 3 for further details).

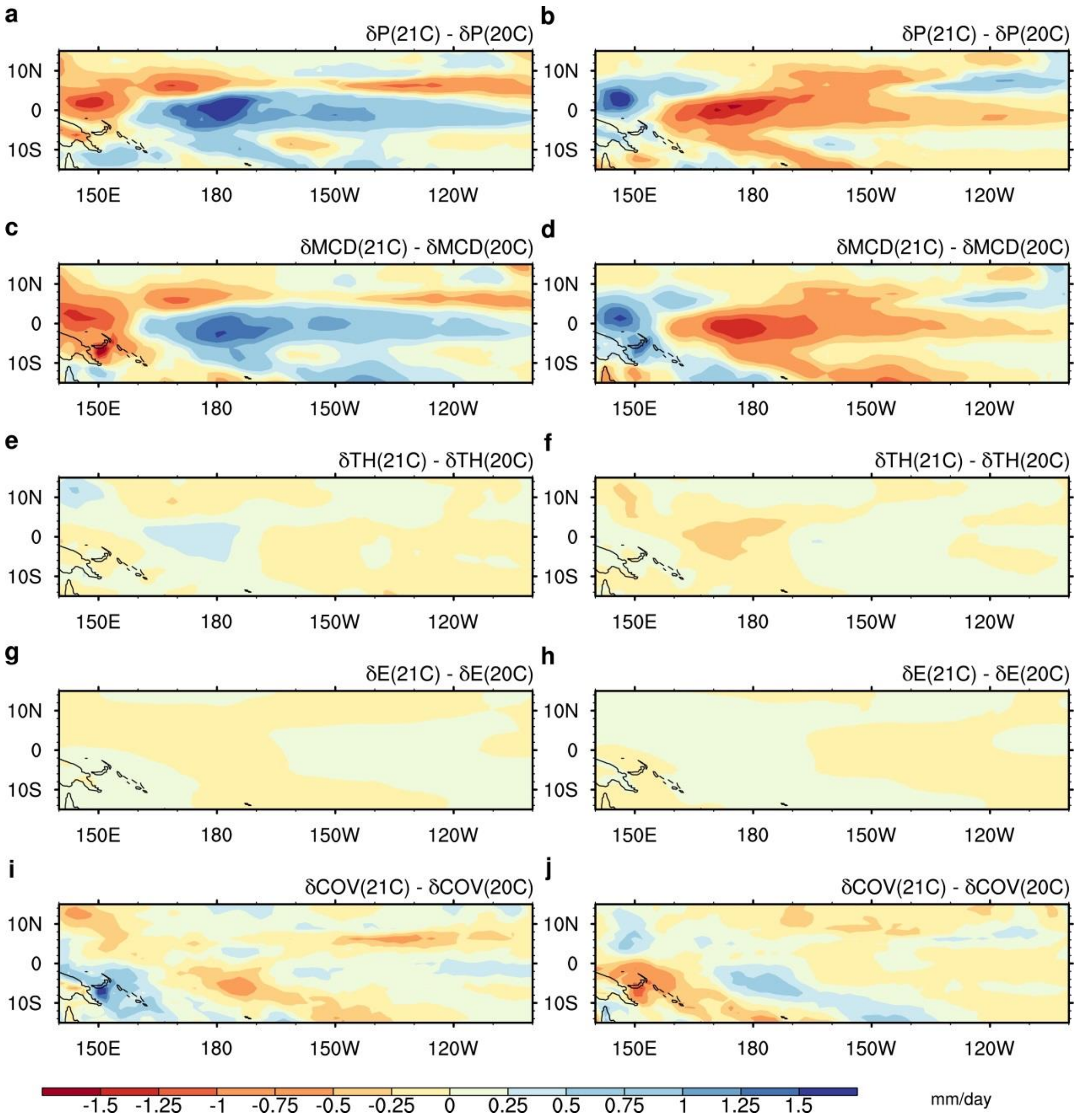

Supplementary Figure 2 | Spatial structure of the change in precipitation in the AGCM, together with contributions to this change from the dynamic (MCD, c, d), thermodynamic (TH, e, f), covariant (COV, g, h), and evaporative (E, i, j) components. Changes during both El Niño (left panels) and La Niña years (right panels) are presented. Changes in the precipitation anomalies are generally dominated by changes in the dynamic component. Maps were generated using the NCAR Command Language Software (Version 6.1.2) 2013, Boulder, Colorado: UCAR/NCAR/CISL/TDD, (http://www.ncl.ucar.edu/).

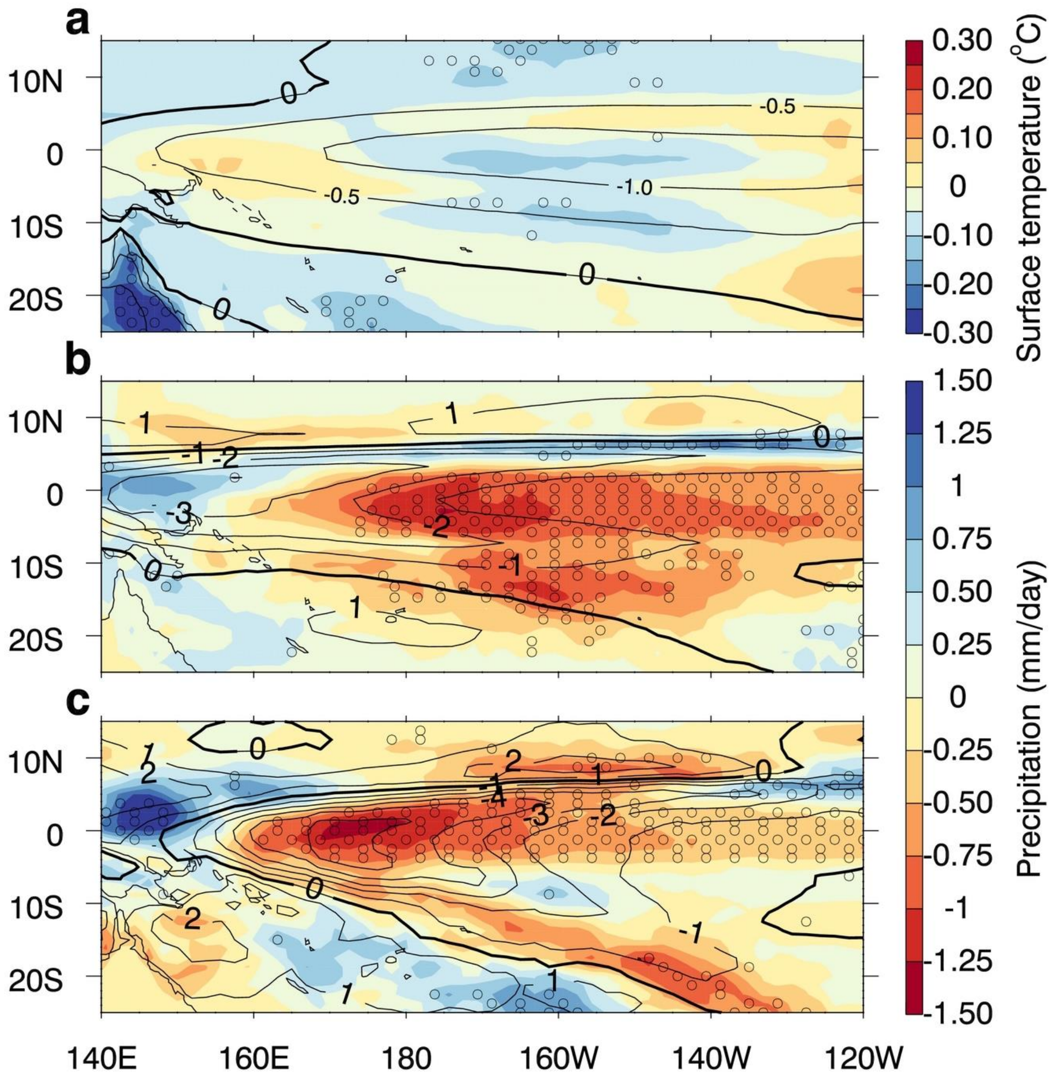

Supplementary Figure 3 | The impact of global warming on the spatial structure of La Niña-driven anomalies. MMM differences in La Niña-driven surface temperature (**a**) and precipitation (**b**) anomalies in the CMIP5 climate models (L21C relative to pre-industrial, RCP8.5). (**c**) The impact of global warming on La Niña-driven precipitation anomalies in the AGCM (under SRES A2) with no change at all in the La Niña-driven SST anomaly. Maps were generated using IDL Version 8.2.3 (see caption to Figure 3 for further details).

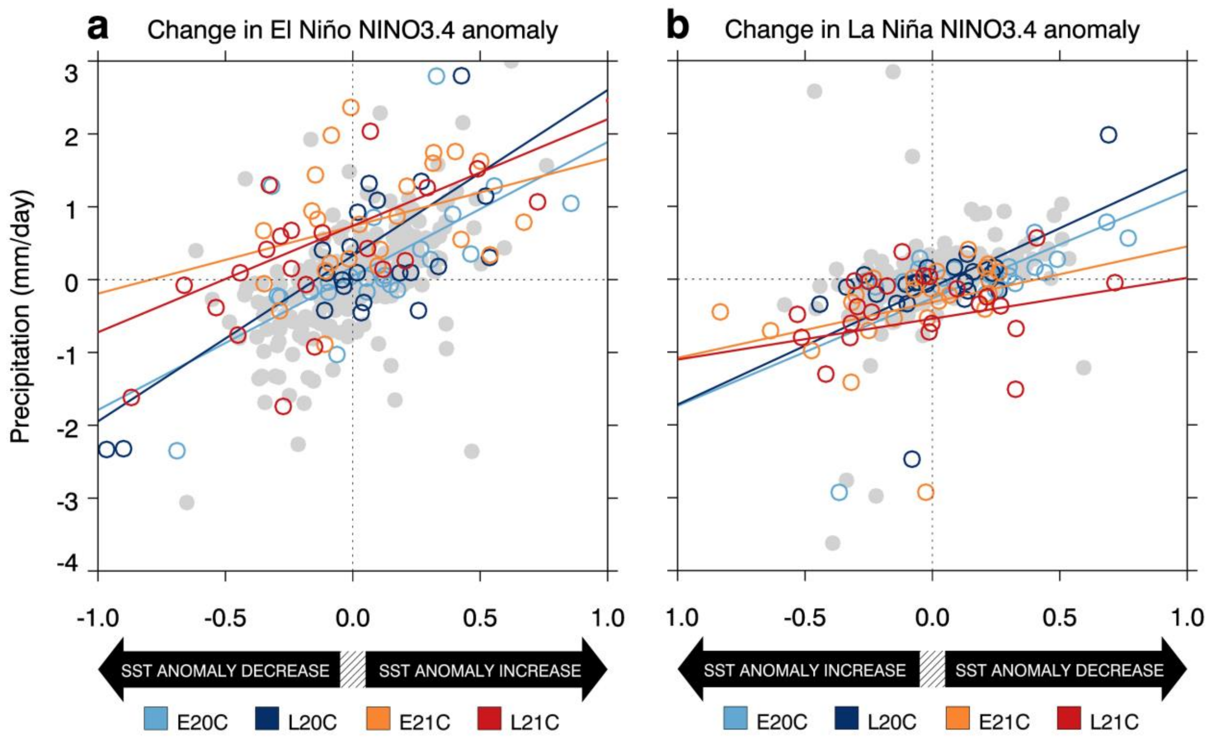

Supplementary Figure 4 | Scatter plots showing changes in NINO3.4 precipitation anomalies (y-axes) and SST anomaly changes (x-axes) relative to the pre-industrial period in each model. a, El Niño, **b**, La Niña. The line-of-best-fit for the changes in all of the models was then determined for each period (i.e. E20C etc.) relative to all ten 50-year pre-industrial periods. One of the ten different lines-of-best-fit for each period is depicted in this figure. The y-intercept indicates the change in precipitation that can occur in the absence of any change in SST anomaly. This provides an estimate of the non-linear contribution to the precipitation anomaly change. The ten individual values of these non-linear contributions to La Niña and El Niño NINO3.4 rainfall anomalies are depicted in Figs 4c and 4d, respectively.

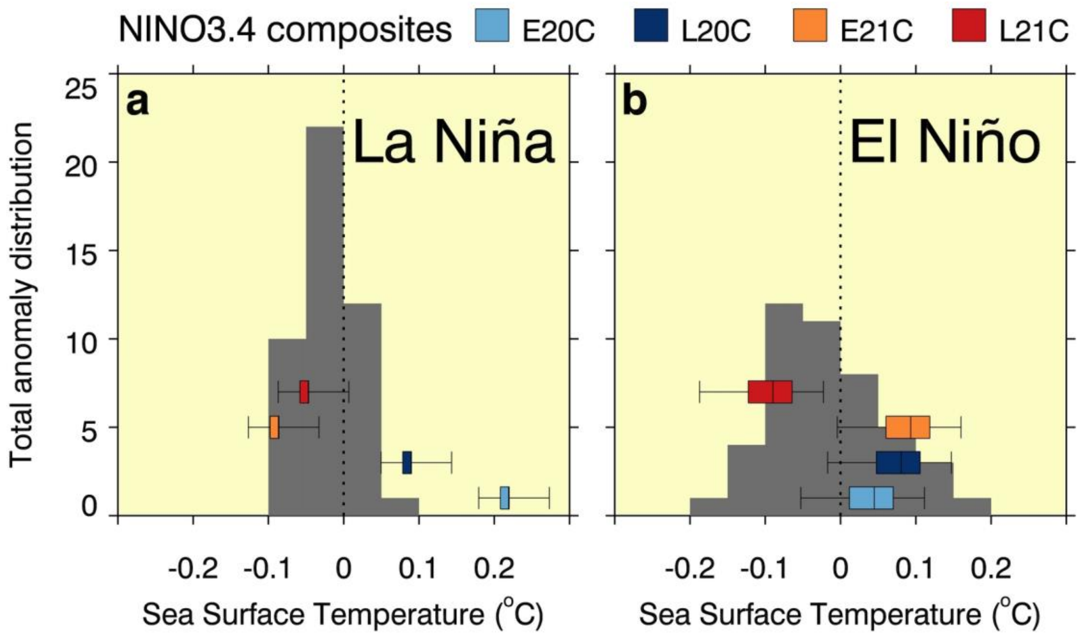

Supplementary Figure 5 | Relative Frequency Distributions of changes in NINO3.4 SST anomaly. a, La Niña, **b**, El Niño. All changes are relative to ten different pre-industrial periods. The grey bars represent the distribution of changes between the ten pre-industrial periods. The grey bars therefore represent apparent changes arising from internal climate variability. The boxplots represent the changes for E20C (light blue), L21C (dark blue), E21C (orange), L21C (red) relative to the ten pre-industrial values. The whiskers indicate the minimum and the maximum change, the boxplot the 25th and 75th percentile and the median is indicated by a vertical line.

Supplementary Table 1 | Models used in this investigation and the forcing applied to them. Models with 500 years of pre-industrial scenario were selected. Twenty-four models were forced using both historical forcing (HIST) and forcing under the RCP4.5, RCP8.5 scenarios from the CMIP5 archive[21]. Most of the results described are based on analysis of the 24 models. One subset is also considered: the 20 models for which simulations under RCP2.6 forcing are also available.

Model	Pre-industrial	HIST	RCP4.5	RCP8.5	RCP2.6
**ACCESS1-0**	X	X	X	X	
**ACCESS1-3**	X	X	X	X	
**bcc-csm1-1**	X	X	X	X	X
**BNU-ESM**	X	X	X	X	X
**CanESM2**	X	X	X	X	X
**CCSM4**	X	X	X	X	X
**CESM1-BGC**	X	X	X	X	
**CMCC-CMS**	X	X	X	X	
**CNRM-CM5**	X	X	X	X	X
**CSIRO-Mk3-6-0**	X	X	X	X	X
**FGOALS-g2**	X	X	X	X	X
**FGOALS-s2**	X	X	X	X	X
**FIO-ESM**	X	X	X	X	X
**GFDL-CM3**	X	X	X	X	X
**GFDL-ESM2G**	X	X	X	X	X
**GFDL-ESM2M**	X	X	X	X	X
**GISS-E2-R**	X	X	X	X	X
**HadGEM2-ES**	X	X	X	X	X
**IPSL-CM5A-LR**	X	X	X	X	X
**MIROC5**	X	X	X	X	X
**MPI-ESM-LR**	X	X	X	X	X
**MPI-ESM-MR**	X	X	X	X	X
**MRI-CGCM3**	X	X	X	X	X
**NorESM1-M**	X	X	X	X	X
**TOTAL**	**24**	**24**	**24**	**24**	**20**

Supplementary Table 2 | Multi-model mean (MMM) frequency of major disruption (Ω_R), change and % change in MMM(Ω_R), number of models in each subset, and number of models showing increases, decreases and no change, and the associated p-values. All changes and % changes are relative to pre-industrial (P). Values are provided for the MMM of all 24 models and for three 21st century scenarios: RCP2.6, RCP4.5 and RCP8.5. P_{sign} and P_{rank} are estimated p-values. See Methods for further details.

Forcing	Nm	Ω_R	$\Delta\Omega_R$	$\%\Delta\Omega_R$	down	No change	up	*Psign*	*Prank*
**Control**	24	1.12	-	-	-	-	-	-	-
**E20c**	24	1.23	0.11	10	9	1	14	0.21	0.82
**L20c**	24	1.47	0.35	31	5	2	17	0.01	0.04
**RCP2.6 E21c**	20	1.32	0.29	26	5	1	14	0.04	0.07
**RCP2.6 L21c**	20	1.43	0.40	36	3	0	17	0.00	0.01
**RCP4.5 E21c**	24	1.48	0.36	32	7	1	16	0.05	0.49
**RCP4.5 L21c**	24	1.43	0.31	28	8	1	15	0.11	0.04
**RCP8.5 E21c**	24	1.73	0.61	54	4	1	19	0.00	0.00
**RCP8.5 L21c**	24	1.47	0.35	31	8	2	14	0.15	0.01

Supplementary Table 3 | Multi-model mean (MMM) frequency of major disruption (Ω_D), change and % change in MMM(Ω_D), number of models in each subset, and number of models showing increases, decreases and no change, and the associated p-values. All changes and % changes are for relative to pre-industrial (P). Values are provided for the MMM of all 24 models and for three 21st century scenarios: RCP2.6, RCP4.5 and RCP8.5. P_{sign} and P_{rank} are estimated p-values. See Methods for further details.

Forcing	Nm	Ω_D	$\Delta\Omega_D$	$\%\Delta\Omega_D$	down	no change	up	*Psign*	*Prank*
**Control**	24	1.11	-	-	-	-	-	-	-
**E20c**	24	1.40	0.29	26	6	2	16	0.03	0.01
**L20c**	24	1.42	0.31	28	6	2	16	0.03	0.01
**RCP2.6 E21c**	20	1.54	0.62	56	3	2	15	0.01	0.00
**RCP2.6 L21c**	20	1.60	0.68	61	4	2	14	0.02	0.00
**RCP4.5 E21c**	24	1.73	0.62	56	5	1	18	0.01	0.01
**RCP4.5 L21c**	24	2.27	1.16	104	6	1	17	0.02	0.00
**RCP8.5 E21c**	24	2.11	1.00	90	3	1	20	0.00	0.00
**RCP8.5 L21c**	24	2.51	1.40	126	5	1	18	0.01	0.00
